# Extracellular Vesicles from Adipose Tissue—A Potential Role in Obesity and Type 2 Diabetes?

**DOI:** 10.3389/fendo.2017.00202

**Published:** 2017-08-18

**Authors:** Xuan Gao, Carlos Salomon, Dilys J. Freeman

**Affiliations:** ^1^Institute of Cardiovascular and Medical Sciences, University of Glasgow, Glasgow, United Kingdom; ^2^Exosome Biology Laboratory, Centre for Clinical Diagnostics, University of Queensland Centre for Clinical Research, Royal Brisbane and Women’s Hospital, The University of Queensland, Brisbane, QLD, Australia; ^3^Faculty of Pharmacy, Department of Clinical Biochemistry and Immunology, University of Concepción, Concepción, Chile; ^4^Maternal-Fetal Medicine, Department of Obstetrics and Gynecology, Ochsner Clinic Foundation, New Orleans, LA, United States; ^5^Mater Research Institute-University of Queensland, Translational Research Institute, Woolloongabba, QLD, Australia

**Keywords:** adipocytes, extracellular vesicles, adipose-derived mesenchymal stem cells, differentiation, insulin resistance, non-alcoholic fatty liver disease

## Abstract

Adipose tissue plays a key role in the development of insulin resistance and its pathological sequelae, such as type 2 diabetes and non-alcoholic fatty liver disease. Dysfunction in the adipose tissue response to storing excess fatty acids as triglyceride can lead to adipose tissue inflammation and spillover of fatty acids from this tissue and accumulation of fatty acids as lipid droplets in ectopic sites, such as liver and muscle. Extracellular vesicles (EVs) are released from adipocytes and have been proposed to be involved in adipocyte/macrophage cross talk and to affect insulin signaling and transforming growth factor β expression in liver cells leading to metabolic disease. Furthermore EV produced by adipose tissue-derived mesenchymal stem cells (ADSC) can promote angiogenesis and cancer cell migration and have neuroprotective and neuroregenerative properties. ADSC EVs have therapeutic potential in vascular and neurodegenerative disease and may also be used to target specific functional miRNAs to cells. Obesity is associated with an increase in adipose-derived EV which may be related to the metabolic complications of obesity. In this review, we discuss our current knowledge of EV produced by adipose tissue and the potential impact of adipose tissue-derived EV on metabolic diseases associated with obesity.

## Introduction

Over the last few decades adipose tissue and adipocyte function has been under extensive study due to their central role in energy homeostasis, obesity, and diabetes (1, 2). The discovery of adipokines has led to the recognition of the key role of adipose tissue secretory products in mediating the consequences of excess adipose tissue accumulation and its wider role in metabolism. More recently, adipose tissue secretion of extracellular vesicles (EVs) and their potential role in the regulation of metabolism and the development of insulin resistance (IR) and type 2 diabetes has come under investigation. This article will review the data available on the nature of the EV produced by the component parts of adipose tissue and their potential local and remote end-organ effects.

Extracellular vesicles are spherical vesicles with an outer lipid bilayer which are released from almost all living cells from bacteria to multicellular organisms (3). EVs are classified according to their size and the pathway by which they were produced (i.e., endocytic or plasma membrane). EVs are grouped by size and origin as exosomes (~40–100 nm), microvesicles (~100–1,000 nm), and apoptotic bodies (~1,000–5,000 nm). Microvesicles and apoptotic bodies are formed directly *via* blebbing of the plasma membrane, whereas exosomes are produced *via* an endocytic pathway (4). It is difficult to classify EV as there is currently a lack of specific EV markers and there are no internationally accepted definitions of EV. EV may be isolated by a variety of different methods, the most common being differential and buoyant density gradient ultracentrifugation, gel filtration chromatography or other size separation techniques, flow cytometry, or by precipitation using polymers or antibodies (5). EVs play an important role in intercellular communications. With a wide range of inhibitory and stimulatory effects, EV can influence a variety of cell functions, including cytokine production, cell proliferation, apoptosis, and metabolism (6). These effects are mediated by the content of EV including RNA (mRNA, miRNA, and other RNAs), protein, and lipids (3). The distribution of EV is widespread, and EV can be isolated both *in vivo* (mainly from body fluids, such as plasma/serum, urine, cerebrospinal fluid, saliva, etc.) and *in vitro* (from cell-conditioned media). The composition and function of EV derived from adipose tissue is poorly understood but of major interest due to the central role of obesity in type 2 diabetes mellitus (T2DM).

## Adipose Tissue Structure and Function

### Types of Adipose Tissue

Human white adipose tissue is distributed throughout the body with the main depots classified as subcutaneous adipose tissue and visceral adipose tissue (7). Eighty percent of white adipose tissue is located in the subcutaneous compartment and up to 10–20% is located in the visceral compartment, mainly around the mesentery and omentum (7). There are also small quantities of adipose tissue located around blood vessels (perivascular adipose tissue) and in liver, muscle, joints, and bone marrow. While subcutaneous adipose tissue functions predominately benignly as a storage depot for excess fatty acids, visceral adipose tissue is more closely linked to the adverse metabolic and inflammatory profile observed in individuals with obesity and IR (8–10). Brown adipose tissue stores are substantial in rodents but in humans are mainly only found in infants or in adults who have undergone cold adaptation (11). Brown adipose tissue promotes non-shivering thermogenesis *via* the expression of uncoupling protein 1 in its mitochondrial membranes and may have an important role in energy homeostasis (12). White adipose tissue can be induced to express some of the features of brown adipose tissue and the resultant adipocytes are termed beige (13).

### Cellular Composition of Adipose Tissue

**Figure 1:**
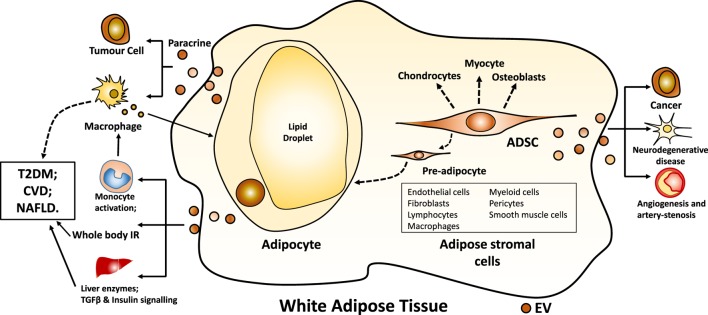
White adipocyte and ADSC-derived EV. Abbreviations: EV, extracellular vesicle; ADSC, adipose tissue-derived mesenchymal stem cells; IR, insulin resistance; TGF, transforming growth factor; T2DM, type 2 diabetes mellitus; CVD, cardiovascular disease; NAFLD, non-alcoholic fatty liver disease.

Adipose tissue comprises adipocytes and adipose-derived stromal cells (Figure 1). Adipocytes are the main cell type in adipose tissue. Excess calories, as fatty acids, are stored in lipid droplets within adipocytes in the form of triglyceride. In the postprandial period, newly formed, smaller adipocytes more avidly take up free fatty acids released from circulating triglyceride in plasma lipoproteins by lipoprotein lipase resulting in the production of larger adipocytes (14). Adipose stromal cells comprise pre-adipocytes, endothelial cells, fibroblasts, lymphocytes, macrophages, myeloid cells, pericytes, smooth muscle cells, and mesenchymal stromal stem cells (15). Adipose stromal cells support the proliferation and the differentiation of pre-adipocytes to adipocytes *in vivo* and *in vitro* and secrete a variety of cytokines and growth factors exerting potential paracrine effects (15). adipose tissue-derived mesenchymal stem cells (ADSC) are multipotent and can differentiate into adipocytes, osteoblasts, chondrocytes, and myocytes (16).

### Adipose Tissue Expansion

When individuals become obese, their excess calorific intake is stored in the form of triglycerides within the adipocytes of white adipose tissue. If there is insufficient capacity in mature adipocytes, new adipocytes are formed from pre-adipocytes in order to increase storage capacity (17). The formation of adipocytes (adipogenesis) occurs in two phases (18). The first phase of white adipose tissue adipogenesis is committed to differentiation and involves the production of committed white pre-adipocytes from mesenchymal stem cells. Once committed, pre-adipocytes lose their multi-potency and can only differentiate into adipocytes or proliferate. Terminal differentiation to form mature white adipocytes results in the characteristic appearance of the mature adipocyte containing one single lipid droplet that occupies almost all the space within the cell. Similarly, brown adipose tissue differentiation comprises a committed differentiation step followed by a terminal differentiation step (18).

In some individuals, there appears to be a limited ability to produce mature adipocytes from pre-adipocytes (hyperplasic adipocyte expansion) and instead excess fatty acids are stored in existing mature adipocytes leading to an increase in their size (hypertrophic expansion) (19). Larger adipocytes tend to be more dysfunctional, and they become insulin resistant resulting in increased lipolysis due to resistance to the anti-lipolytic effects of insulin (8). Failure of angiogenesis and provision of an adequate blood supply to hypertrophic adipocytes leads to necrosis, macrophage infiltration into adipose tissue and inflammation and adipokine release. “Spillover” of fatty acids unable to be retained in subcutaneous adipocytes leads to an increase in the visceral fat compartment and eventually flux of fatty acids into ectopic sites, stored as intracellular lipid droplets in tissues, such as liver and the pancreas. The formation of ectopic fat is closely linked to the development of IR and T2DM, and individuals with limited adipocyte expandability, such as South Asians, are at increased risk of type 2 diabetes (20).

## EVs from Adipocytes

### Composition of Adipocyte EVs

Extracellular vesicle production has been studied in whole adipose tissue explants (subcutaneous and visceral) (21, 22), isolated adipocytes, *in vitro* differentiated adipocytes (21) and in ADSC (see later). Kranendonk et al. (21) characterized EV isolated by differential ultracentrifugation from *ex vivo* subcutaneous and visceral adipose tissue explant cultures and from human adipocytes differentiated *in vitro* from Simpson Golabi Behmel Syndrome (SGBS) pre-adipocytes (23). Both adiponectin-positive and adiponectin-negative EVs were produced by whole adipose tissue, as determined by flow cytometry and could be separated by differential ultracentrifugation (21). The authors suggested that adiponectin-positive EV came specifically from adipocytes, rather than stromal cells (21). Differentiated human adipocytes produced EV containing the presumed adipose-specific markers FABP-4 and adiponectin as well as a number of inflammatory adipokines, including MIF, TNFα, MCSF, and RBP-4 (21). Adipokine profile differed between subcutaneous adipose tissue EV and visceral adipose tissue EV with concentrations of IL-6, MIF, and MCP-1 significantly higher in visceral adipose tissue EV compared to those from subcutaneous adipose tissue (24). The exosome miRNA profile from lean and obese individuals was compared and 55 differentially expressed miRNA were found (25). The differentially expressed miRNAs identified were predicted to regulate the transforming growth factor (TGF)β-signaling and wnt/β-catenin signaling pathways (25). A differential EV proteomic profile has also been observed between obese diabetic and obese non-diabetic rats (26).

Extracellular vesicle production by adipocytes has also been studied in the mouse 3T3-L1 pre-adipocyte cell line which can be differentiated to mature adipocytes (27). EV concentration across a size range of 0–1,000 nm was measured by nano-tracking analysis and concentrations of all particle sizes were more than threefold higher prior to adipogenesis (27). Higher particle concentrations were particularly observed for particles less than 300 nm. Lipid composition of EV was different after adipogenesis with higher membrane phosphatidyl serine content and a higher proportion of arachidonic acid (27). There was no change in EV protein marker expression (CD9, CD63, TSG101, and Alix) or PPARγ protein content after differentiation but FABP-4 and PREF-1 levels decreased while adiponectin content almost doubled (27). 3T3-L1 adipocyte exocytosis has been shown to be stimulated by cAMP *via* a PKA-independent pathway and this was regulated *via* both calcium-dependent and -independent processes (28). Exosome release from 3T3-L1 cells was increased by the long chain omega-3 fatty acid docosahexaenoic acid (29). Because of potential hypoxia in adipose tissue resulting from adipocyte hypertrophy, the effect of hypoxia on 3T3-L1 differentiated adipocytes exosome production was assessed (30). 3T3-L1 adipocytes exposed to hypoxia had a different exosome proteomic profile compared to control adipocyte exosomes (30). In particular, exosomes produced under hypoxic conditions were enriched in enzymes involved in *de novo* lipogenesis and these exosomes were able to promote lipid accumulation in recipient 3T3-L1 cells.

Murine brown adipocytes produce exosomes and their production is increased by cAMP treatment (cAMP is the second messenger induced by cold exposure and β-adrenergic stimulation) (31). Beige, but not white, adipocyte exosome production was increased nearly 11-fold by treatment with cAMP (31). When mouse whole adipose tissue was exposed to cold, exosome production was also increased (31). miRNA profiling of the exosomes produced by murine brown adipocytes identified miR-92a as a specific marker that was downregulated on cold exposure (31). Feeding mice a high fat diet induced whitening of brown adipose tissue and an increase in serum miR-92a levels, whereas cold exposure was associated with decreased serum miR-92a levels. In lean humans whose brown adipose tissue activity was assessed by labeled glucose uptake, there was a negative correlation between brown adipose tissue activity and serum miR-92a levels (31). Furthermore, in 10 human subjects exposed to cold acclimatization for 10 days, serum miR-92a abundance was decreased and the change in miR-92a levels correlated with the change in brown adipose tissue activity.

### Functions of Adipose Tissue-Derived EV

Extracellular vesicles derived from adipose tissue may play a role in the paracrine cross talk between adipocytes and macrophages (Figure 1). EVs secreted from both subcutaneous and visceral adipose tissue explants and from SGBS-derived adipocytes were able to promote the differentiation of primary monocytes into macrophages. These macrophages had the same cytokine secretory profile as macrophages found in human adipose tissue, reflecting a mixed M1/M2 phenotype (21). Adiponectin-positive EVs were much more effective than adiponectin-negative EVs in effecting monocyte differentiation as were EVs derived from visceral, rather than subcutaneous tissue explants (21). Conditioned medium was collected from macrophages exposed to adipose tissue EV. When human adipocytes were then exposed to this conditioned macrophage medium, insulin signaling as assessed by Akt phosphorylation was inhibited in the adipocytes (21). Differentiated 3T3-L1 adipocytes when stressed by exposure to palmitic acid produced microparticles which could act as chemo-attractants for monocytes and primary macrophages (32). Purified murine adipose tissue exosome-like vesicles when injected back into mice were taken up by peripheral blood monocytes which then differentiated into macrophages secreting TNFα and IL-6 (33). When exosome-like vesicles produced by ob/ob mice, which have a high RBP-4 content, were injected into wild-type mice, the recipient mice developed IR (33). This response was less marked in TLR4 knockout mice (33). Taken together these data provide evidence that EV produced by adipocytes may communicate with immune cells and influence whole body IR.

Kranendonk et al. looked at the effects of subcutaneous and visceral adipose tissue EV on insulin signaling and expression of genes involved in gluconeogenesis in a hepatocyte cell line (HepG2) and in myotubes differentiated from a myoblast cell line (C2C12) (24). There was a mixed response to individual adipose tissue EV preparations in hepatocytes. Adipose tissue EV from the majority of individuals inhibited Akt phosphorylation while adipose tissue EVs from a few individuals stimulated Akt phosphorylation. There was no effect of either subcutaneous or visceral adipose tissue EV on myotube insulin signaling. After exposure of hepatocyte cells to subcutaneous and visceral adipose tissue EV, there was a negative association between Akt signaling and glucose-6-phosphatase gene expression (24). Exosomes produced by visceral adipose tissue explants from obese humans can integrate into HepG2 cells and hepatic stellate cell lines (22). Gene expression profiling of HepG2 cells exposed to these exosomes indicated dysfunctional extracellular matrix regulation in HepG2 cells and dysregulation of the TGFβ signaling pathways in both HepG2 and hepatic stellate cell lines (22). The authors proposed that *in vivo* these changes may induce liver fibrosis and may link obesity to non-alcoholic fatty liver disease (22). Similar observations were only made at high doses of visceral adipose tissue exosomes from a single lean individual but it is hard to draw conclusions regarding differences between exosomes from lean and obese individuals from this single experiment (22).

Adipocytes can be found in the tumor microenvironment and have been shown to promote tumor progression (34). Obesity is a risk factor for melanoma and its malignant progression. In a study of human subcutaneous adipocyte exosomes isolated by differential ultracentrifugation (35), there was a correlation between adipose tissue exosome shedding and donor BMI. When used at equal concentrations, exosomes from overweight and obese donors increased melanoma migration more than exosomes from lean individuals in a dose-dependent manner (35). This effect was thought to be mediated *via* fatty acid oxidation as inhibition by etomoxir reversed the effect (35). Interestingly, exosomes produced by mature 3T3-F442A adipocytes were found by mass spectrometry to contain an abundance of proteins involved in lipid metabolism, particularly those involved in fatty acid oxidation (35).

### Adipocyte EV and Whole Body Metabolic Status

Circulating EV levels have been linked to cardiovascular disease (CVD), diabetes, and non-alcoholic fatty liver disease (33, 36, 37) but the extent to which circulating EV derived from adipose tissue are involved is uncertain. Circulating EVs positive for CD9 and adiponectin have been isolated from the plasma of patients with aortic aneurysm (24) raising the possibility that adipose tissue may communicate with other organs *via* EV release. Obesity is a risk factor for CVD, type 2 diabetes and non-alcoholic fatty liver disease and may be associated with either higher EV release from adipose tissue stores or release of EV with a different functionality (22, 25, 26, 35) with downstream metabolic consequences for end organs involved in these diseases (Figure 1). The difficulty is being confident about which specific markers identify EV from adipose tissue. As discussed above, cellular experiments have suggested adiponectin, FABP-4, and RBP-4 to be adipocyte EV-specific markers. Proteomic profiling of extensively purified human plasma exosomes identified the presence of PPARγ, a key transcription factor in adipocyte differentiation (38). However, since this protein can also be expressed in vascular endothelial cells and immune cells, this cannot categorically identify adipose tissue as a source of plasma EV. Similarly for adiponectin, it has been suggested that exosomes only account for a minor proportion of the total adiponectin secreted from 3T3-L1 cells (29) and, thus, it may not be a reliable marker for plasma EV. Others using mouse whole body models have suggested that perilipin A can act as an adipocyte EV marker (32, 39). The lack of definitive data on this issue makes it difficult to interpret experiments on circulating EV proposed to be from adipose tissue and the following data should be viewed with caution.

The number of visceral, but not subcutaneous, *ex vivo* adipose tissue-derived EV per gram of fat, quantified by flow cytometry, correlated with whole body HOMA-IR (but not body mass index or hsCRP levels) in patients (*n* = 11) with aortic aneurysm from whom the adipose tissue biopsies were collected (21). In a similar study, subcutaneous adipose tissue EVs produced from 16 individuals were inversely correlated with waist circumference and presence of metabolic syndrome, while visceral adipose tissue EV correlated positively with plasma liver enzymes (24).

Circulating adipose microparticles, as determined by the presence of perilipin A, from ob/ob mice resulted in activation of monocytes in the circulation and in adipose tissue of wild-type mice (32). Perilipin A-positive EVs are higher in mice with diet-induced obesity and in humans with metabolic syndrome (39). In humans, circulating perilipin A-positive EV could be reduced by a 3-month low calorie dietary intervention. Using FABP-4 as a marker of adipocyte-derived circulating exosomes, the change in adipocyte-derived exosome miRNA profile a year after gastric bypass surgery was assessed (40). Changes in miRNA profile that were predicted to regulate the insulin signaling pathway were observed and the degree of change in miRNA profile linked was correlated with both the change in IR as assessed by HOMA and the change in plasma levels of branched chain amino acids (40).

In a larger study of 1,012 patients with vascular disease, plasma EV were isolated by precipitation and characterized by multiplex immunoassay (41). Microvesicles containing the proteins cystatin C and CD14 have previously been shown to be associated with atheroma plaque size, and increased risk of CVD morbidity and mortality (42). The relationship between levels of these cystatin C positive and CD14-positive EV with subcutaneous and visceral adipose tissue thickness and metabolic disease prevalence and incidence was assessed. High EV-cystatin C levels were associated with high plasma hsCRP and low HDL-cholesterol (41). HDL-cholesterol was positively associated with EV-CD14 levels (41). EV-cystatin C levels were associated with increased odds of metabolic syndrome (OR 1.57; 95% CI 1.19–2.17); however, there was no relationship between EV-cystatin-C levels and obesity or whole body IR (41). By contrast, EV-CD14 were associated with reduced hazard of developing type 2 diabetes (HR 0.84, 95% CI 0.75–0.94) over a 6.5 years follow-up (41). Again it is difficult to know whether these EV risk markers are actually produced by adipose tissue. EV-cystatin C and EV-C14 are released by monocytes, endothelial cells, and platelets; but while CD14 adipose tissue gene expression and cystatin C protein secretion (43) is increased in adipose tissue from obese humans, there is no direct evidence that these proteins are secreted in EV from adipose tissue.

## Adipose Tissue-Derived Mesenchymal Stem Cells (ADSC)

There is great interest in adipose tissue as a source of stems cells for regenerative medicine for use in cancer and other diseases. Beneficial effects of such treatments may result from paracrine effects of ADSC mediated by EV. Exosomes produced by ADSC isolated from human adipose tissue have been shown to contain small RNA species predominately miRNA and snoRNA with some evidence that tRNA species are enriched (44). It appears that the contents do not merely reflect the source cellular content but that some RNA species are preferentially released (44). There is evidence that the protein secretory profile of ADSC differs from individual to individual and that raises the possibility that ADSC comprise a heterogeneous population of cells of functionally different subtypes and may produce corresponding EV (45). There are also data to suggest that functional aspects of ADSC differ from stem cells derived from other tissues (46). EVs from ADSC have been considered as a delivery route for delivering therapeutic miRNA to diseased cells.

### ADSC EV in Cancer

Adipose tissue-derived mesenchymal stem cells were isolated from abdominal fat from patients undergoing neoplastic urological surgery and from participants without cancer (47). miRNA profiling of exosomes from these ADSC showed that, in both cancer and non-cancer patients, some exosome and ADSC miRNA content was similar. However, there did appear to be selective enrichment of some miRNA (let-7-a-1, miR-21, and miR-1260b) into exosomes (47). ADSC transfected with a miR-122 expression plasmid secreted exosomes containing miR-122. When the miR-122-containing exosomes were added to hepatocellular carcinoma cells, the cells became sensitive to chemotherapeutic drugs (48). Furthermore, intra-tumoural injection of transfected ADSC EV exosomes increased the effectiveness of an anti-cancer agent on hepatocellular carcinoma in an *in vivo* mouse model (48). ADSC exosomes have also been shown to promote migration of a breast cancer cell line (MCF7) (49).

### ADSC EV in Vascular Disease

One study has compared EV produced by ADSC isolated from subcutaneous and visceral adipose tissue in obese and non-obese individuals (50). There was no difference in EV number or size between ADSC derived from non-obese or obese subjects (50). The content of VEGF and MMP-2 (markers of EV angiogenic potential) was lower in EV derived from the subcutaneous and the visceral compartment of obese individuals compared to those derived from the same compartments in non-obese individuals (50). In addition, the content of miR-126, but not that of miR-130A, (both pro-angiogenic miRs) was also reduced in EV produced by ADSC from obese individuals (50). These data suggesting that EV from obese ADSC, either from subcutaneous or from visceral depots, may have reduced pro-angiogenic potential were confirmed by *in vitro* experiments showing that EV from obese ADSC had reduced ability to induce migration and tube formation in cultured endothelial cells (50). Palmitic acid treatment could induce the anti-angiogenic EV changes *in vitro* and this suggests that increased circulating fatty acids in human obesity may influence the function of ADSC (50). ADSC EV secretion was stimulated by platelet-derived growth factor (PDGF) and changed EV protein composition enhancing their angiogenic properties (51). PDGF-stimulated ADSC were able to promote vessel formation when injected subcutaneously in Matrigel into mice (51). ADSC microvesicles have also been shown to increase migration and tube formation by human umbilical vein endothelial cells (52). The active agent carried in the microvesicles was thought to be miR-31 that inhibited factor-inhibiting HIF-1 (52).

Restenosis of vein grafts is a particular problem with coronary artery bypass surgery. ADSC have been used to try and reduce neointimal hyperplasia in vein grafts. Human ADSC were shown, by *in vitro* cell culture and in a mouse model, to reduce vein graft neointima formation by inhibiting vascular smooth muscle cell proliferation and migration and reducing macrophage migration and inflammation (53). ADSC also inhibit the activation, differentiation, and proliferation of T cells (54). In a porcine model of metabolic syndrome and renal artery stenosis, treatment with autologous EV from ADSC resulted in decreased renal inflammation and increased kidney blood flow and glomerular filtration rate (55). Others have found that a combined treatment of ADSC plus ADSC-derived exosomes had superior ability, compared to either one alone, to protect the kidney from acute ischemia–reperfusion injury in a rat model (56).

### ADSC and Neurodegenerative Disease

ADSC are also of interest for the treatment of neurodegenerative disorders such as amyotrophic lateral sclerosis (57). Murine ADSC exosomes protect motor neurone-like NSC-34 cells from oxidative damage increasing their viability (57) and similarly human ADSC exosomes protect neurones against glutamate-induced damage (58). Others have shown in mice *in vitro* that ADSC nanovesicles and microvesicles prevented apoptosis in neuronal cells and increased remyelination in cerebellar slices demyelinated using lysophosphatidyl choline (59). Again EV have been considered as a delivery vehicle with therapeutic implications for neurodegenerative disease. ADSC secreted exosomes containing neprilysin, a major β-amyloid peptide degrading enzyme, and the exosomes were capable of delivering this protein to a neuroblastoma cell line (N2a) resulting in decreased secreted and intracellular β-amyloid peptide (60). Therefore, there is therapeutic potential for such EV in Alzheimer’s disease. Furthermore, exosomes from ADSC have been shown to have therapeutic potential in an *in vitro* mouse neuronal cell model of Huntington’s disease (61).

## Conclusion

Adipose tissue can produce EV from both adipocytes and ADSC (Figure 1). The early data presented above indicate that the adipose depot from which adipocyte EV are derived, and the relative state of obesity of the donor may influence the function of such EV. We are still at the first stages of research. No definitive marker of adipocyte-derived EV has yet been agreed upon nor has a consensus characteristic EV cargo been identified. This makes it difficult to interpret data on circulating EV in obesity and metabolic diseases such as type 2 diabetes. Although the data do suggest that circulating EV profiles change in obesity and metabolic disease, it is not yet clear whether EVs act merely as a marker of disease or whether they play a functional role in tissue to tissue communication. Initial cell culture and animal model experiments do suggest that there is the potential for communication between adipocytes and immune and liver cells *via* EV. Adipose tissue has also proved to be a source of stem cells and EV produced by ADSC appear to have potential roles in cancer, angiogenesis, vascular disease, and neurodegenerative disease, both as a direct effector to promote or prevent pathogenesis and as a delivery system to target beneficial miRNA to cells as a therapeutic option.

## Author Contributions

XG reviewed the literature, wrote the first draft, and created the figure. CS reviewed the article for accuracy as EV expert and edited final draft. DF reviewed the literature, wrote the final draft, and made editorial changes.

## Conflict of Interest Statement

The authors declare that the research was conducted in the absence of any commercial or financial relationships that could be construed as a potential conflict of interest.
